# First wave of psychological impact and associated factors in hospitalized COVID-19 patients; cross sectional study in supra tertiary hospital in Thailand

**DOI:** 10.1192/j.eurpsy.2023.1685

**Published:** 2023-07-19

**Authors:** J. Na Bangxang, P. Wongkulab

**Affiliations:** 1 Psychiatry; 2 Internal medicine, RAJAVITHI HOSPITAL, Bangkok, Thailand

## Abstract

**Introduction:**

The Severe Acute Respiratory Syndrome Coronavirus (COVID-19) pandemic not only impacted on physical but also mental health of the patients. We investigated the prevalence and associated factors of depression, anxiety among hospitalized patients with COVID-19. Rajavithi hospital is a supra-tertiary hospital which was the frontline in the first wave and gets referral SARS CoV-2 cases from other parts of Thailand.

**Objectives:**

We investigated the prevalence and associated factors of depression, anxiety among hospitalized patients with COVID-19.

**Methods:**

A cross-sectional study was designed to evaluate prevalence of depression, anxiety and associated factors among 93 hospitalized COVID-19 patients between 1 July 2020 – 31March 2021. Depression and anxiety were measured with Thai Hospital Anxiety and Depression (Thai HADS).

**Results:**

Prevalence of depression was 5.4 %, prevalence of anxiety was 2.2 % Depression was associated with age greater than or equal to 60 years old (p-value = 0.028) and requiring supplemental oxygen therapy (p-value = 0.021).

**Conclusions:**

This study shows low prevalence of depression and anxiety in hospitalized patients with COVID-19. Depression was significantly related with elderly and supplemental oxygen therapy. Elderly is vulnerable to depression during treatment and patients who require supplemental oxygen therapy. Severity of the disease might affect neuroinflammatory responses which can relate to depression. Furthermore, severity of disease puts patients in more isolation or guilt that might lead to depression.

**Disclosure of Interest:**

None Declared

